# Emotional Labor in Dementia Research

**DOI:** 10.1177/10497323241274709

**Published:** 2024-10-17

**Authors:** Catherine Quinn, Alexandra Hillman, Ana Barbosa, Gill Toms

**Affiliations:** 1 Centre for Applied Dementia Studies, 1905 University of Bradford, Bradford, UK; 2 Wolfson Centre for Applied Health Research, Bradford, UK; 3 Wellcome Centre for Cultures and Environments of Health, 3286 University of Exeter, Exeter, UK; 4 School of Medical and Health Sciences, 1506 Bangor University, Bangor, Wales, UK

**Keywords:** research methods;, qualitative research;, quantitative research;, interviews;, stress;, burnout;, well-being;, boundary;, emotion work

## Abstract

The concept of emotional labor refers to the regulation and management of emotions within the workplace. This labor may involve a dissonance between the emotions that are internally felt and the emotions that can be externally expressed. The concept of emotional labor can be applied to the emotional management that occurs during research often when directly interacting with research participants. These emotions can have a positive role in building rapport and enabling the researcher to understand the participant’s world. But equally, it can lead to emotional strain and potentially have a negative impact on researcher well-being. In this paper, we apply the concept of emotional labor to dementia research. While there has been attention paid to ethical issues in dementia research, this has often focused on the impact on the participant and not the researcher. Within this paper, we first draw on the literature to provide an overview of the role of emotional labor in the research context. Within the literature, we identify nine research scenarios where emotional labor might occur within dementia research. We then present three case studies illuminating our experiences of emotional labor within dementia research. These case studies provide illustrative examples of some of the research scenarios identified in the literature. To synthesize the learning from the literature and our case studies, we propose peer-critiqued recommendations for managing emotional labor in dementia research. We conclude by considering the implications for other health researchers.

Health research commonly involves an element of direct interaction with participants, for example, through qualitative interviewing or the administration of questionnaires. Often through these interactions, some form of rapport is established: the researcher learns something about the participant but also shares something of themselves in the process of establishing a connection. While the establishment of rapport is an important part of the research process, it often involves researchers undertaking emotional labor in their interactions with participants, and this can potentially have negative impacts on researchers if they are unprepared for this aspect of their work or if support is not available.

## Concept and Theory

Emotional labor refers to the management of emotions within the workplace (e.g., Hochschild, 2012). The concept recognizes that within some roles, workers may need to regulate their emotions during their work interactions. It recognizes the effort and control that can be needed to “express organizationally desired emotion during interpersonal transactions” (Morris & Feldman, 1996, p. 3). To paraphrase, this involves managing the emotions of someone else, while also working to regulate your own feelings (Mallon & Elliott, 2019). This means that there may be incongruence between the emotions the person is experiencing and the organizationally desired emotion (Morris & Feldman, 1996). Thus, it distinguishes between emotions that are internally felt and the emotions that are externally displayed. There are two levels of “acting” that comprise the emotional labor the person engages with. Surface acting involves displaying the correct emotion (e.g., smiling) without the person changing how they fundamentally feel. In comparison, deep acting involves the person changing their internal feelings to display the emotion that will best facilitate the situation (Huynh et al., 2008).

An idea somewhat related to emotional labor is the assertion that undertaking research can have an emotional impact on researchers. This is the context within which emotional labor occurs. For instance, a researcher is upset by a story shared by a participant (the emotional impact of the research), but they know it would not be helpful to cry in front of the participant and so they use surface acting, not showing that they are upset (the emotional labor). The emotional impact of undertaking research is more often discussed, and when we draw on this literature in this paper, we will identify the emotional labor that this impact may require.

Undertaking emotional labor requires skill (Weeks, 2007), and depending on how it is deployed, emotional labor can have a positive or negative impact on well-being (Martinez-Inigo et al., 2007; Wharton, 1999). Negative impact can result because emotional labor may affect an individual’s ability to differentiate between themselves and their work role, increasing the risk of burnout (Wharton, 1999).

The concept of emotional labor has developed over time. Originally, it was applied to female roles in paid and domestic work. It was also traditionally applied to customer-focused professions. For instance, it has often been applied to occupations that involve contact (normally face to face or voice to voice) with individuals external to the organization and which require the person to manage their own emotions (Dickson-Swift et al., 2009). It has also been applied to those working with healthcare. For example, healthcare workers may feel professionally obligated to limit the expression of their emotion (Brighton et al., 2019; Huynh et al., 2008; Riley & Weiss, 2016). Healthcare workers commonly interact with and manage individuals experiencing negative emotions such as distress, trauma, and anger (Riley & Weiss, 2016). In terms of emotional labor, staff working in palliative care have described having to govern the level of emotion they display in front of patients and their families (Brighton et al., 2019).

Emotional labor has also been applied to non-work contexts, for example, unpaid carers for people with motor neuron disease (Ray & Street, 2006). Ray and Street (2006) described how carers for someone with motor neuron disease felt unable to share their reactions to the symptoms, behavior, or the stress of caring with other family members and the person themselves. It is only more recently that emotional labor has been applied to researchers (e.g., Bergman Blix & Wettergren, 2015). Although researchers are not practitioners providing a service, their involvement with participants often involves a considerable amount of personal interaction (Micanovic et al., 2020). Mallon and Elliott (2019) have used emotional labor as a theoretical framework to conceptualize the emotional impact of sensitive research on researchers. They note, despite this recent application to research, emotional labor has received little attention within the research policy space.

## Framework for the Paper

In this paper, we apply the concept of emotional labor to researchers working in dementia research. This is not the only area of investigation where researchers might need to undertake emotional labor, but it is a pertinent example, and it builds on literature that has explored the impact of researching sensitive topics on the researcher. Dementia is a degenerative brain condition and an area of increasing research activity (Pickett & Brayne, 2019). There has recently been a greater focus on directly hearing from the person with dementia about their experiences (Quinn et al., 2022). It is a subject matter that may elicit emotional reactions for researchers. First, given the large numbers of people living with dementia (World Health Organization [WHO], 2022), many researchers will have some personal experience of people living with dementia and this will heighten emotions. This can be exacerbated by the stigma that can be associated with dementia (WHO, 2022). Second, people with dementia can experience difficulties accessing support (Gauthier et al., 2022) and therefore may seek help and advice from the researcher. Third, research in dementia also often includes family members and care staff, so the researcher will often need to manage multiple (sometime simultaneous) interactions. Fourth, the presentation of dementia, which may include fluctuating awareness (Clare et al., 2011), confusion, unpredictable emotions, and deterioration over time (WHO, 2022), can make it more likely that the research will have an emotional impact on researchers. Given the high potential for emotional impact, many researchers in this field will need to undertake emotional labor at some point to maintain participants’ emotional safety. The deterioration that occurs in dementia over time means that the emotional labor required is likely to change over the course of longitudinal research.

Our approach to this paper was to use a framework of combining the research evidence on this topic with our own personal experiences of undertaking research. The paper comprises three sections, which build upon each other:

1. We provide an overview of the literature on emotional labor in research. Drawing on dementia and other healthcare research, we identify nine scenarios in which emotional labor can occur.
2. We present case studies from our own dementia research structured using Gibb’s reflective cycle (Stice, 1987). These examples illustrate some of the emotional labor scenarios identified from the literature.
3. We present recommendations for researchers working on dementia projects synthesized from the literature and the case studies. These recommendations have been peer critiqued.

In the discussion, we highlight gaps in our knowledge and consider how the learning can inform other areas of health research.

## The Role of Emotional Labor Within Research

In 2001, Hubbard et al. shared the opinion that the role of emotional labor in research is often “undervalued and at worst, ignored all together” (Hubbard et al., 2001, p. 122). One potential reason for this is a persistent perception within academia that it is inappropriate to admit to feeling emotional in carrying out research as it could indicate a lack of rigor and professionalism (Dickson-Swift et al., 2009). Up to now, the predominant focus has been on the impact of the research process on participants. Less attention has been paid to the short- and long-term emotional impact on the researchers (Emerald & Carpenter, 2015) and how researchers navigate these impacts within their work. Despite this, the role of emotional labor has started to be recognized in the research process alongside other research exploring the emotional impact of the research process on researchers. Typically, these studies have focused on the emotional labor of undertaking qualitative research (e.g., Hoffmann, 2007) or fieldwork (e.g., Hubbard et al., 2001), drawing on the researchers’ personal experiences. Other studies have explored the emotional labor involved in researching sensitive topics (e.g., Hanna, 2019) or in co-production (Faulkner & Thompson, 2023). In this section, we draw on the findings of studies that have specifically explored emotional labor as well as studies that have looked more broadly at the impact of the research process on the researcher.

There is published evidence suggesting that collecting data around sensitive topics can be emotionally intense for researchers and that emotional labor in these contexts can have harmful consequences for the researcher (Allmark et al., 2009; Dickson-Swift et al., 2006; McGarrol, 2017; Silverio et al., 2022). One identified consequence is emotional strain. This can arise when researchers need to manage their display of emotion using either surface or deep acting (Bergman Blix & Wettergren, 2015; Dickson-Swift et al., 2009). Hubbard et al. (2001) provide the example of a researcher feeling angry or annoyed with a participant but being unable to show or express this. There is some discussion in the literature that the consequences of such emotional labor may vary according to different career stages. For instance, those new to research may be at more risk of neglecting the impact that emotional labor may have on them (McGarrol, 2017) and therefore may be more likely to experience negative consequences. Writers in this area have also identified that emotional labor can involve the management of positive emotions. Deep acting in this context can help the researcher strengthen their relationship with the participant and enter into and more fully understand the participant’s world (Emerald & Carpenter, 2015).

The literature additionally highlights that different types of data collection can involve different forms and degrees of emotional labor. There is one example indicating that emotional labor can occur in the context of collecting quantitative data such as when meeting with participants to complete standardized measures (Kennedy et al., 2014). In relation to qualitative data, Hoffmann (2007) describes the challenges of open-ended/unstructured interviews as there can be drift into unintended areas. Some authors have suggested emotional labor is more difficult when directly collecting data from participants, for example, through face-to-face or voice-to-voice contact (Hoffmann, 2007). However, other research indicates that emotional labor can also occur when working with written data such as posts on online forums or diaries (Hanna, 2019; Scott, 2022). Researchers may be undertaking these tasks in shared offices or need to present and discuss this data in team meetings, in supervisory meetings, and when reporting their activities to funders. All these scenarios might require surface acting on the part of the researcher. The findings from Micanovic et al. (2020) suggest that surface acting can be particularly emotionally taxing during data analysis activities because there is a forensic focus on the participant’s accounts. Others have similarly highlighted that transcribing interviews in environments where emotions cannot be shown might also be taxing as the researcher has more time to dwell on the information (Dickson-Swift et al., 2007). We suggest the researcher may find they are more aware of their emotional reactions during transcription than during the interview when they will have been dividing their attention between what was being said, responding to the participant, and keeping the data collection on track. There is evidence that the process of transcribing can elicit reactions that need emotional labor to manage in a professional workplace. For instance, professional transcribers have reported that they feel emotionally attached to the data, wanting to know how the participants’ stories ended (Lalor et al., 2006).

## Emotional Labor Within Dementia Research

The issue of emotional labor has not been directly addressed within dementia research. A systematic review of research on ethical issues in dementia research (Götzelmann et al., 2021) identified issues that could have an impact on the participant or research project but did not identify any topics relating to emotional labor on the part of the researcher conducting the research. Papers have been published on specific ethical issues in conducting dementia research (e.g., Beuscher & Grando, 2009; Carmody et al., 2015) and when involving people living with dementia in developing and delivering research (e.g., Miah et al., 2020; Webb et al., 2020). While these papers have not directly addressed the issue of emotional labor, some of the topics identified could be considered to involve emotional labor.

A paper by Beuscher and Grando (2009) drew on their own personal experiences of conducting qualitative research with people with dementia, identifying some of the challenges faced, such as obtaining consent. Similarly, a paper by Heggestad et al. (2013) identified ethical challenges experienced in a qualitative study with people with dementia, such as the disclosure of the dementia diagnosis. A further paper by Carmody et al. (2015) drew on the evidence-base to discuss issues around conducting qualitative research with people with dementia: for example, divulging personal information. Finally, a review by Novek and Wilkinson (2019) of including people with dementia in qualitative research highlighted issues such as the importance of relationships, rapport building, and dealing with distress. Therefore, there is some indication that there are issues occurring within dementia research that could be contributing to emotional labor.

Drawing on dementia research literature and other healthcare research, we identify nine scenarios where emotional labor may arise:

1. Given the cognitive difficulties experienced by people with dementia, ethical and practical issues around exploring capacity to consent have received much discussion (Beuscher & Grando, 2009; Carmody et al., 2015; Götzelmann et al., 2021). Consent is often an ongoing process and is re-confirmed at each research encounter (Dewing, 2008; Murphy et al., 2015). It is surprising therefore that scarce consideration has been given to the emotional labor that researchers might need to undertake in this process. Getting to know the individual and building rapport can help in ascertaining the individual’s decision-making capabilities (Murphy et al., 2015). Yet, in our experience, by building this rapport it can be difficult for the researcher to deal with a situation where the person wants to participate, yet the final determination is that they do not meet the eligibility criteria. For example, studies may only include people who have capacity to consent (Götzelmann et al., 2021). After establishing rapport, it can be hard for researchers to feedback that the individual is ineligible. In this scenario, it may not be helpful for the researcher to display their sadness or disappointment with this outcome, and they may need to employ surface acting to express the emotion that they believe will best support the participant.
2. As part of the consent process, dementia researchers will often need to work with people supporting the individual with dementia, who may be practitioners and/or family members (Götzelmann et al., 2021; Novek & Wilkinson, 2019). Dementia often changes the power dynamic of the relationship between people with dementia and family members providing care (Quinn et al., 2009, 2015). Therefore, these people often have a role in facilitating the participation of the person with dementia. These situations can also impact emotionally on researchers and therefore require emotional labor. Kennedy et al. (2014) identified that researchers can find carers or care staff challenging to engage with. They suggest this can occur when a researcher feels that the carer or staff member is expressing opinions that are disempowering or diminishing the voice of the person with dementia. We propose that in such scenarios, it would be understandable for researchers to wish to challenge viewpoints they perceive as problematic. They may feel irritated, angry, or aggrieved on behalf of the person with dementia. In most conceivable circumstances, it will be inappropriate for the researcher to express their sentiments and deep or surface acting will be needed to maintain a good relationship with all the individuals involved. In addition, the family member or member of care staff and person with dementia may have differing views and this conflict can be difficult to manage in the research context, as the researcher needs to validate both viewpoints. This will require at least surface acting so that the researcher can support all the individuals involved.
3. People with dementia vary in awareness and understanding of the condition (Quinn et al., 2018), and this awareness can fluctuate (Clare et al., 2011). Similarly, their family members may have a limited understanding of dementia (Quinn et al., 2015, 2017, 2019). Dementia is a life-limiting condition, and the term dementia can arouse strong emotional reactions and can be “contested” (Novek & Wilkinson, 2019). Although these points are discussed in the dementia literature, the impact on researchers has not been written about. Researchers working with contested language, people with fluctuating awareness and understanding, and where terms can elicit strong reactions are likely to feel anxious in interactions with participants, at least sometimes. Fluctuations in awareness mean that the researcher might need to engage in different emotional labor each time they meet with the participant or even at different points during a single meeting. We propose that this is likely to be draining for the researcher, as they can never “relax” as there might be sudden changes in awareness. Anxiety will have an emotional impact on the researcher and requires emotional labor, so that the anxiety is not transmitted to others and interactions are as comfortable as possible for participants.

Researchers may experience other emotions when awareness and understanding fluctuate. They may find themselves in conversations where the perceptions expressed are contrary to their own understanding. Hubbard et al. (2001) identified that researchers can hear things that they find distressing, and this distress may persist after the encounter has ended having a lasting negative impact. Considering emotional labor, we would add that when disagreement in views arises, the researcher may need to employ deep acting so that rapport is maintained.

4. A further demand on dementia researchers is that little or fluctuating awareness can increase the risk that people with dementia will experience distress at some point in the research process. It has been suggested that researchers may feel psychologically uncomfortable collecting “data” with people who are distressed, even if the person wishes the research activity to continue (Bloor et al., 2007). Managing negative emotional reactions can be difficult when working with someone with cognitive impairment as they may not recall what triggered the emotional response. Hubbard et al. (2001) identified that participants’ emotional responses can directly impact the emotions of the researcher, and others have recognized that it can be difficult for researchers to limit their own emotional reaction (Dickson-Swift et al., 2009). In our experience, identifying the initial signs of distress in people with dementia can also be difficult, particularly if there is impaired verbal communication (Novek & Wilkinson, 2019). The researcher will need to work harder to spot changes in emotion and initiate appropriate, supportive responses.

We further suggest that concern about identifying distress and responding appropriately may cause the researcher stress and anxiety. Handling these emotionally charged experiences can require considerable emotional labor, especially when the expression of participants’ emotion can be unpredictable and the researcher feels they have a lack of control over the situation (Micanovic et al., 2020). Even if these scenarios are well planned, Mitchell (2011) highlights that they remain challenging “in the moment.” The emotional labor can leave researchers feeling anxious or guilty about how they managed (Mitchell, 2011).

5. It is important for researchers to build rapport with participants to gain their trust. An important aspect of building rapport is the sharing of information to make a personal connection. Based on our experience, if a researcher has personal experience of dementia, they must carefully consider what impact sharing their personal experience may have on participants and if it is to their benefit. Sometimes, sharing personal experience will support participants; at other times, it may cause distress. For instance, a participant in the early stages of dementia may not be ready to hear the researcher’s experience of the latter stages. Furthermore, researchers with personal experience of dementia may find that hearing the experiences of others triggers their own emotions and memories. Both these scenarios will require emotional labor so that supportive emotions are expressed. In the literature, this has been considered when people with dementia act as peer researchers on projects. When these peer researchers are involved in data collection and analysis, there needs to be consideration of the emotional impact this might have on individuals (Waite et al., 2019). Research exploring emotional labor in people bringing lived experiences into mental health research has highlighted that emotional distress can occur (Faulkner & Thompson, 2023). We would add that there also needs to be consideration of how these peer researchers can manage these emotional impacts “in the moment” so that encounters remain supportive for participants.
6. Developing rapport in dementia research can result in role boundaries becoming blurred (Novek & Wilkinson, 2019). Role boundary challenges can seem innocuous. As Novek and Wilkinson (2019) report, it is not unusual for participants and their family members to ask dementia researchers questions about dementia, about treatment options, or about what services are available. In our experience, even these innocuous boundary challenges can require emotional labor for the researcher. Researchers may not feel they have the clinical expertise to answer these questions. If they do have the prerequisite expertise, they will need to gauge how to answer in a manner that is supportive of the asker. They may be reluctant to share the “truth” about an individual’s prognosis or they may feel distressed because they cannot provide the wanted reassurance. Emotional labor needs to moderate how any hesitancy, anxiety, reluctance, or distress is expressed.

The blurring of boundaries can involve other role conflicts where researchers are drawn into tasks that are outside their role. This is noted in the literature when researchers are working in institutional or healthcare settings (Bloor et al., 2007; Johnson & Clarke, 2003). For instance, researchers may be perceived as “extra staff” in these settings (Bloor et al., 2007) and find themselves being expected to take on clinical tasks. As discussed in one of the case studies, the researcher might also find themselves in a position where they feel they need to step in. Feeling compelled to undertake tasks outside one’s role can create irritation, frustration, anxiety, and concern. Researchers will need to manage these emotions and use surface or deep acting so that positive working relationships are maintained. They will also need to use emotional labor to be assertive, when appropriate, so that people receive the care they need and that the integrity of the research is maintained.

7. A further area where boundaries can get blurred is when the process of establishing rapport with a participant merges into forming a friendship. It is clear in the literature that researchers sometimes feel uncomfortable if friendships develop as this can form a perceived threat to professional distance and the boundaries that protect the integrity of the research (Bloor et al., 2007; Dickson-Swift et al., 2007). We have found that if a friendship forms, both parties must consider wider factors that mitigate against it ultimately being a good course of action to continue the relationship. It may be challenging for researchers to take on board these broader considerations when they have found the relationship supportive for their own well-being. Although the emotional impact of these scenarios has had some discussion, the emotional labor this incurs has not. It is conceivable that deep acting may be needed to help guide interactions, as deep acting helps the researcher consider the perspective of the person with dementia. Surface acting may also be needed so that the researcher does not overburden the participant with their own sadness and sense of loss that a friendship cannot continue.
8. Although discussed indirectly, it is recognized that deep acting can have emotional impacts on researchers. Having empathy (a core outcome of deep acting, which considers the other’s viewpoint to try and alter internal emotions) is part of the emotion work in developing and sustaining rapport within research relationships (Watts, 2008). In our experience, building rapport and a relationship with people with dementia can be challenging because they are on a trajectory of decline. If the research is undertaken over a long time period, it can be emotionally difficult to witness this decline. It is noted in the literature that researchers can experience distress when they witness the decline of participants, but believe they need to be emotionally neutral during encounters, not sharing their felt distress with participants (Kennedy et al., 2014). We would add that researchers might also have limited opportunities to share their distress with colleagues or supervisors after such encounters.
9. Ending the relationship at the conclusion of the research can be a further challenge, especially when good rapport has been established (Götzelmann et al., 2021). To elaborate, in our experience, if you perceive that you have been “helping” someone, it is difficult to walk away, especially from participants who are living in tough situations and receiving little support from elsewhere. The emotional impact of this has been explored in the literature. The researcher can feel guilt at “abandoning” their participants (Lalor et al., 2006). They can experience a “lack of closure” as they often do not get to hear how the person is getting on afterward (Greenwood et al., 2015; Hanna, 2019). Of course, sometimes a relationship may end before the research project finishes, for example, if the participant becomes unwell or dies (Watts, 2008). The potential unexpectedness of this may magnify the emotional impact. Again, the emotional labor required in these situations has not been discussed. It will often be unhelpful for researchers to express feelings of guilt in encounters with participants, and they may feel unable to address any lack of closure within supervision. Therefore, most likely, surface acting will be employed to “mask” these feelings.

## Gaps in the Literature

The literature overview highlights the scarce literature on emotional labor in all research fields, including dementia. Where the impact of the research process is discussed, the focus tends to be on the impact on participants. In dementia research, there has been some discussion of the emotional impact of research on researchers (primarily drawn from qualitative research), but how this is managed to maintain supportive encounters with participants and to uphold professional interactions with colleagues has not been discussed. Furthermore, it would be expected that emotional labor occurs throughout the research process, particularly with the emphasis on public patient involvement where the researcher will be interacting with intended beneficiaries from the project inception to dissemination and implementation activities. This is not reflected in the current literature, which focuses exclusively on data collection and analysis tasks.

## Case Studies

Given the dearth of research on emotional labor in dementia research, we now share our own reflections in case studies. Albeit our own experiences, this represents an initial attempt to share some “evidence” about researchers’ experience of emotional labor. These illustrate some of the nine scenarios for emotional labor identified in the literature.

The case studies have been anonymized and any names used are pseudonyms. The case studies were written independently by the researchers using Gibbs’ reflective cycle (Stice, 1987) which provides a framework for examining experiences. The cycle involves stages encompassing a description of experience; feelings about this experience and analysis; and an evaluation of the experience including what was learnt. Prepared case studies were shared and discussed with the lead author. The lead author suggested areas where rewording or further details would improve clarity, and changes were mutually agreed. The team then met and reflected on all the case studies, and this helped inform the initial draft recommendations.

### Case Study 1: Managing Distress

#### Description

Undertaking fieldwork for me has consisted of many conversations with people with dementia and their families—both in the form of guided conversational interviews and in less formal interactions that surround those research encounters. As enriching as these experiences have been, they have also been emotionally demanding. I had planned for the many practical and ethical dimensions of supportively including those living with dementia in research; however, I was much less prepared for the emotions I would feel and the need to manage these emotions in the research encounter.

Interviews often produce a variety of different emotional responses, and these can change throughout the course of an interview. However, when interviewing people with dementia and their families, these sorts of fluctuations in emotion can be less predictable and can be tricky to manage. I often found the unpredictability of emotional responses to be unnerving in not knowing if and how you may inadvertently cause distress. I also had to be alert, to recognize these changes and navigate the best way to respond. There are two specific examples that come to mind that illustrate this emotional labor.

• The first occurred during a visit to undertake an interview with Fred who was in his eighties and living with Alzheimer’s disease and his wife Maggie. Fred and Maggie had lost their son in an accident when he was in his twenties. During the interview, while talking about his previous job, Fred began to describe this tragic event and became tearful and distressed. I suggested that we take a break from the interview, paused the recorder, and handed Fred a tissue. I went to get him a glass of water and we sat quietly for a few minutes, and I asked if he would like to continue. Although we did restart the interview, this sudden recollection of this tragic event did leave a mark. I remembered, in that moment, thinking back to a conversation I’d had with one of our Patient Public Involvement members who has Alzheimer’s disease, who had said to me that people who have dementia may not remember what has been said during a conversation, but “feelings remain.” Using deep acting, I tried my best to end the interview with Fred on some more positive and affirming topics, but even though the conversation moved on, the emotion seemed to stay with him, and I felt guilty that this conversation had triggered it. I did not believe it would be helpful to Fred or Maggie if I shared my guilt with them (this could re-kindle the memories) and so I used surface acting to show positive emotions when our encounter ended.
• The second example involved me interviewing a mother and daughter. Judith, who had vascular dementia, lived with her daughter. Judith was extremely talkative and was happy to share many aspects of her life. However, while talking about her children when they were young, Judith began to talk about her ex-husband who she described as a cruel man who had since passed away. As she described him, she became increasingly agitated and angry and began to speak as though he was a presence in her life now. I sought to change tack, to move the conversation in a different direction, but this proved increasingly difficult, with Judith becoming more distressed. I then suggested that we take a break for a drink. The general feeling of anger seemed to permeate the encounter and had to be carefully negotiated in the remainder of the interview. I felt it was important not to display the discomfort or anxiety that this anger was causing me and used surface acting to ensure my body language, tone, and emotional expression were calm and measured throughout.

#### Feelings and Analysis

I felt quite anxious during these moments but felt I had to stay calm. I was concerned that Fred and Judith were experiencing negative emotions that seemed to linger even after the conversation had moved on. I felt worried about how best to navigate these emotions, to both acknowledge them and provide support, but also to help steer the conversation toward topics that might engender more positive feelings. Although emotional labor helped me manage both situations, I was left with lingering emotions and questions following interviews of this kind: could that distress have been avoided? Could I have managed it better? These questions and the worry they carry can stay with you for some time.

#### Evaluation

In both these situations, I sought to respond to the situation in the moment, to read the reactions of the participants and to use emotional labor to act in ways that felt supportive. This work can have an emotional impact. Researchers who work with people with dementia often undergo a process of second guessing themselves, wondering what the “right” thing to do is. However, is there a “right” way in any given situation?

### Case Study 2: Navigating Power Dynamics

#### Description

For a while, my research work involved going out to talk to people with dementia and their family members face to face in the community. This was part of a mixed-methods research study that was evaluating a new group-based psychological intervention for people with dementia. The intervention was for people with early symptoms of dementia, so for many of the people I was meeting, they had just embarked on the journey of “living with dementia.” One of the conditions for eligibility was that both members of the dyad—the person living with dementia and the family carer—took part in completing evaluative assessments (both quantitative and qualitative).

One of the frequently arising situations that was hard to manage emotionally was when “dyads” had competing perspectives/understandings, wishes, and needs as my emotions could be pulled in different directions and it was often not possible to reach a resolution that suited both parties. This presented in various ways over the 2 years of the project. Two representative examples are as follows:

• Once when I had been invited by a spousal carer to discuss the project and initiate recruitment conversations, I found their partner was adamant that they had no interest. A conversation around their concerns and answering questions about the study did not alter their mind; in fact, they were very clear they wanted nothing to do with any research! This necessitated explaining to the carer, why we could not go any further with the study, despite the carer’s palpable desire to “get involved.” The emotional labor involved ensuring that my body language and tone did not lead the person with dementia to feel pressure to change their opinion while ensuring I showed some degree of empathy to the spousal carer, acknowledging her disappointment.
• An even more uncomfortable situation arose when a person with dementia who participated in the first stage of the study (a qualitative interview) decided not to proceed to Phase 2 (the intervention phase of the project), despite the wishes of his wife. His wife was distressed by this as she had pinned a lot of hope on the intervention improving her support network and social situation. Again, the emotional labor was to ensure that the emotions I displayed would support both individuals and not increase the discomfort of either the person living with dementia or the spouse.

Relationship dynamics could also elicit a strong emotional reaction in me that I needed to manage in the moment. Tensions (sometimes evidently longstanding) were often aired during my visits. This could include negative feelings being vented, and unhelpful beliefs or assumptions about the person with dementia being expressed in their presence (e.g., “he can remember when he wants to”). On occasions, the person with dementia could be talked about as if they were a child. Sometimes, it was hard to “hear” the voice of the person with dementia or ascertain what they really thought or felt. This was the case when questions were answered on their behalf or when they seemed to acquiesce (typically with a smile and a shrug) to the dominant narrative presented by their carer. The emotional labor involved deep acting, considering the perspective of the spouse and person with dementia to consider what might be influencing their reactions and behavior. This normally led me to feel less angry, irritated, and frustrated (meaning it was less likely I would display these emotions). Deep acting also informed how my emotional response in the encounter might support both individuals.

#### Feelings and Analysis

I think my overriding feeling was one of helplessness in these scenarios. This was helplessness tinged with anger though, often I think reflecting the feelings of the people I was meeting. I felt helpless because I couldn’t meet the contradictory wishes of both people. The carer’s expressions of exasperation often seemed to come from a belief that their own needs were not being recognized, and as I needed to primarily be led by the person with dementia, I was repeating this pattern. The exasperation of the people with dementia I met was often more muted, but my own feelings of anger often related to how the dynamics within a relationship seemed to be robbing the person with dementia of some of their dignity, and I felt powerless to change this trajectory.

#### Evaluation

My guiding principle when undertaking emotional labor was to “tread lightly” in the relationship I had been invited into for a brief period, so that I would hopefully leave no footprints behind to add to the challenges already accumulating. This meant listening, signposting, when I could answer questions answering these honestly, but trying to express neutral emotions so as not to be drawn into the relationship dynamics. Essentially, emotional labor allowed me to remain neutral even though this did not always sit comfortably with my emotions that were urging a greater advocacy role. I often managed these unresolved emotions (the impact of the emotional labor) through informal debriefing with colleagues who could provide reassurance that what I had done to manage these situations in the moment was appropriate. This reassurance and the opportunity, to share my feelings and to hear that other researchers had faced and felt similar things, allowed me to achieve a form of closure.

### Case Study 3: Blurring of Boundaries

#### Description

Much of my research has been in care homes. My journey started with a PhD focused on improving person-centered dementia care in these settings, which involved implementing and evaluating a multisensory stimulation program for people with advanced dementia. Among others, I was responsible for recruiting, obtaining people’s consent, and collecting and analyzing quantitative and qualitative data from observations. Some of these were participant observations—for example, when I joined the group involved in the multisensory stimulation sessions—but most were non-participant observations—for example, when I watched people’s daily routine from a distance.

When researching people with dementia in care homes, I found that maintaining role boundaries was difficult. Through observations, I was able to capture valuable, rich, and in-depth information about the individual, relationships, and the environment. However, I also became aware of practices I was trying to challenge, and knowing exactly how to act in those situations was particularly challenging. Two examples are as follows:

• Observing a disregard of people’s psychosocial needs. A common issue was a lack of or inappropriate communication with people with dementia (e.g., people not being consulted before making decisions or being rushed to perform an activity). This often led me to feel concerned or angry, but it was often not appropriate to express this through my words, demeanor, and emotional reactions.
• Other times, the participants themselves disclosed practices that were not meeting their needs. Often in these occasions, there was a blurring of boundaries where I was considered part of the care team rather than being viewed as a researcher and was given access to information that went beyond the scope of my work. Again, I often felt out of my comfort zone, but displaying this discomfiture would not have supported participants.

#### Feelings and Analysis

Often, when I captured things that were, in my view, a threat to people’s psychosocial needs, I found myself feeling deeply uncomfortable, powerless, and frustrated. I faced a dilemma between reporting findings and being a non-judgmental outsider aiming to maintain a positive relationship with everyone. Gaining and maintaining access to care homes and having the support of staff is often a lengthy but vital process for successful research. If relationships are compromised, through expressing emotions at the wrong moment or in a way that is non-supportive, then the research might be jeopardized. The emotional labor was to express more neutral emotions throughout my encounters, but this labor had a negative emotional impact. I often felt that by being mute I was not helping participants but using them as merely objects of scrutiny.

#### Evaluation

I was fortunate to be in a situation where I was able to approach my supervisors and colleagues to gain support and advice. This debriefing together with reflective practice helped to maintain the right perspective. It helped me to be become more aware of the context of the care home environment and recognize there are many factors that might be influencing the level of care provided. Over time, I understood that suspending all judgments and just watching, listening, and thinking about what I had seen until after the observation was over was effective. In essence, supervision helped me employ deep acting as well as surface acting. Therefore, I developed the approach of just “letting go” unless there was a clear need to act, for example, someone’s safety was directly threatened. Through using more deep acting, the emotional impact of the emotional labor was lessened. It also made me realize that if my guiding principle was to keep the well-being of the participant center stage, this also meant clarifying expectations at regular intervals throughout the research. Paying close attention to participants’ expectations about me and my role helped me to set clear boundaries.

## Recommendations

Based on our learning from the literature and our reflections on the case studies, we now synthesize recommendations for supporting researchers to undertake emotional labor activities, particularly in the field of dementia research (see Table 1). The relevance of these recommendations will vary depending on the research environment (e.g., in someone’s home) and methods for data collection, that is, online or face to face. These recommendations were co-drafted by the authors in a meeting. The recommendations were then reviewed by each author independently and refinements offered. The iterated draft was shared with eight researchers who were peers of the authors. These researchers all worked in dementia research but were at different career stages (details provided in the Acknowledgments). These individuals reviewed the list of recommendations and provided verbal or written feedback. Based on this feedback, a final iteration of the recommendations was created.

**Table 1.** Recommendations to Support Researchers With Emotional Labor.

Emotional Labor at Different Stages of the Research Process
When planning research • Plan with the mindset that it is normal to experience a range of emotions when doing research with people with dementia. Consider what emotional labor might be needed and how you will mitigate any negative impacts from this labor. • In supervision and/or team meetings, have conversations about how to respond to various “what if” scenarios. For instance, how might you display supportive emotions when someone is keen to participate but they do not meet inclusion criteria? How could you respond professionally if you observe something you find distressing? This preparation should help with emotional labor if these scenarios occur. • If the research will involve working with health or care staff, take time to understand the organizations involved and who needs to provide permissions. This understanding could assist you to deep act when you feel frustration about organizational barriers to the project. It will also help you think through how your research will benefit staff and the organization and so make a compelling case to the organization and staff members about why they should support the project. • If you have personal experience of dementia, think through how much information about this it may be comfortable and appropriate to share with participants. This will help with emotional labor as you will have anticipated what to share before the situation arises. • To mitigate the potential negative impacts of emotional labor (e.g., feelings of guilt), find out where you can signpost participants for information and support. Having a guide to dementia that you can offer to participants if they ask questions can also help you respond to information requests while maintaining your role boundaries. • Guilt around taking up people’s time can be a common emotional impact of research endeavors. Consider ways to acknowledge the individual’s contribution through inviting them to an end of study event or through a token of appreciation if funds allow. At a minimum, you should plan to share findings with participants. • Anticipate that emotional labor may have negative impacts. Leave sufficient time between data collection visits so that you have space to process and release emotions you could not express during the visit. Try not to arrange data collection visits for late in the day, so there is no debrief or “recovery” time before the end of the working day. Factoring in this time also means you will be able to manage any arising issues, for instance, if you need to arrange further support for a participant who is distressed. • Plan what to do after each data collection visit to process and “move on” from any negative emotional impacts or the strain of the emotional labor undertaken. It can be easy to ruminate over emotional labor activities to an extent that impairs well-being. It could be helpful to plan an evening activity that will distract from ruminative thoughts, a relaxation routine, or a physical activity that may help with stress.
When undertaking the research
• Due to the fluctuating awareness of the person with dementia, you may find yourself in situations where you are unsure whether the participant knows who you are or why you are there. Be alert to verbal and visual indicators, and if it seems appropriate check in that they are still happy talking with you. This can prevent the need for emotional labor. If necessary, propose a short break. This gives you time to consider what to do, reducing the strain involved in emotional labor activities. • Similarly, the need for emotional labor related to blurred boundaries can be mitigated by setting clear expectations about your role when meeting participants. Being able to signpost elsewhere for advice and support can reduce the emotional impact of not being able to respond fully to participants’ requests. • Likewise, emotional labor may be needed less if appropriate boundaries are instigated from the outset. For example, provide participants with a work phone number (if possible) rather than your personal mobile number. Having an automated message about work hours and response times can help manage participants’ expectations. • Use deep acting to help you respond positively if family members want time with you. They may be concerned about what you are doing and may be under significant stress from caring. Making time to listen to family members can help you understand their viewpoint and helps build rapport, which might help overcome challenges that need to be addressed. For instance, after hearing their concerns, it might be more possible to arrange a one-to-one interview with the person with dementia. • If you are in a situation where you are becoming emotional (i.e., it is hard to undertake emotional labor effectively), see if you can orchestrate a short break. For instance, you could ask to use the bathroom. Alternatively, change track in the conversation. If you can, center yourself (and help calm strong emotions) by taking a few deeper breaths, taking a small drink of water, and diverting your attention from your distress to something concrete in the immediate environment. For example, you could notice a pattern in the furniture or tune into background noises. It can also be “ok” to acknowledge to the participant that you are feeling emotional and say you need to take a moment before continuing. • Time to debrief with a colleague, mentor, or supervisor after data collection is important. Identify who you can talk to or perhaps set up a mutual support group.
Toward the end of the research
• Take time to reflect on your research interactions. Sometimes, keeping a reflective journal can help. This will help process feelings that perhaps you were unable to express “in the moment” and help you consider what you have learnt from your emotional labor. • Mitigate the need for emotional labor by preparing the participant for the end of your work together. Remind participants of when final meetings will be (e.g., I will visit you once more). Discuss with participants how they will spend the extra free time they will have at the end of the project. You might signpost them to available activities, such as local groups or other research opportunities. This can help manage researcher guilt. You may also wish to send a card to participants at the end of the study to thank them for their contributions and to share how you have enjoyed the time you spent together. This card will also serve as a reminder for the participant that the project has now finished. • Also prepare yourself for the end of the study to help mitigate emotional labor during “ending” tasks. It can be helpful to reflect on whether there is an aspect of the project work that contributed to your well-being that could be maintained in another way. For instance, voluntary roles can offer companionship and are a good way to continue to “make a difference.”

## Discussion

In this paper, we have synthesized information about when and how emotional labor can occur in dementia research from the literature and from our research experience. We have suggested recommendations based on our learning from the literature overview and reflections on our case studies. These recommendations have been critiqued by peers.

In summary, while the emotional impact of the research process on participants is commonly discussed, the impact on the researcher tends to be a neglected area. There is some acknowledgment in the literature of the emotional impact of undertaking dementia research, but the emotional labor this often necessitates is seldom discussed. Undertaking effective emotional labor is important in supporting the well-being of participants but can have emotional impacts for researchers. The lack of discussion in the literature about this raises the risk of researchers entering research encounters unprepared for the emotion work involved. It may also mean that they are not able to access appropriate support to help them manage the negative impacts of emotional labor, particularly if they feel the emotions they are experiencing are “unprofessional.” We identify significant gaps in our understanding about how researchers working with people with dementia undertake emotional labor at various stages of the research process, how they prepare for this aspect of their work, and how they manage any impacts of these activities. Our development of key areas in which emotional labor may occur in processes of conducting research with people with dementia and sharing case studies is an initial contribution to the literature in this area.

This paper adds to the current exploration of emotional labor in dementia research. Through this paper, we have considered emotional labor at all stages of the research process incorporating data analysis, meetings with colleagues and supervisors, and public involvement activities. The extant literature primarily focuses on data collection activities. The literature also predominantly focuses on qualitative methods, whereas we have considered implications for quantitative methods as well. We also share our first-hand experiences of undertaking emotional labor to represent what this can involve and to consider the potential impacts on researchers. The recommendations provide a first set of guidance for dementia researchers who have not considered this aspect of their role.

Although drafted with dementia researchers in mind, the recommendations transfer to researchers working in other fields, such as health research where emotionally supporting participants who may be vulnerable is part of the research process. Many researchers will have lived experiences of health conditions such as cancer. Some health conditions have similarities with dementia in terms of the researcher having to deal with unpredictable behaviors and emotions, and some conditions can lead to periods of altered awareness (e.g., epilepsy and diabetes). So, the scenarios we have discussed in which the need for emotional labor is likely may occur in other health research fields. Furthermore, blurring of professional boundaries can occur in any research, especially qualitative health research where researchers themselves may be healthcare professionals. We believe the learning shared in this paper has wide transferability and the potential to positively impact both researcher well-being and the quality of research. Emotional labor has not been extensively covered in other sensitive research topics (Mallon & Elliott, 2019), and researchers working in other areas will likely have received little training or support related to this facet of their role. Given the growing interest in exploring sensitive topics through qualitative methods (Mallon & Elliott, 2019), the need to consider emotional labor is only going to increase.

In conclusion, this paper has highlighted the importance of considering and planning for the impact of emotional labor in all stages of the research process and in ensuring that researchers are appropriately supported to cope with this emotion work. By doing so, this will reduce the risk of negative impact on the researcher and ensure that we can continue to support participants and undertake important health research.
